# A dual-branch multi-modal deep learning framework for non-destructive evaluation of intramuscular fat in sheep

**DOI:** 10.1038/s41598-025-32208-2

**Published:** 2025-12-18

**Authors:** Qingqing Ling, Haizheng Yu, Chengguang Yue, Jie Kang, Aili Maimaiti, Zhonghui Li, Jiang Di, Mingjun Liu, Long Liang, Wenrong Li

**Affiliations:** 1 College of Mathematics and Systems Science, Xinjiang University, Urumqi, China; 2 Key Laboratory of Animal Biotechnology of Xinjiang Institute of Animal Biotechnology, Xinjiang Uygur Autonomous Region Academy of Animal Science, Urumqi, China; 3 Key Laboratory of Genetics, Breeding & Reproduction of Grass-Feeding Livestock, Ministry of Agriculture, Urumqi, China; 4 Beef + Lamb New Zealand Genetics, Dunedin, New Zealand; 5 Institute of Animal Science, Xinjiang Uygur Autonomous Region Academy of Animal Science, Urumqi, China

**Keywords:** Sheep Quality Assessment, Intramuscular Fat (IMF)Prediction, Multimodal Deep Learning, Computer Vision, Dual-Branch Network, Computational biology and bioinformatics, Engineering, Mathematics and computing

## Abstract

The content of Intramuscular Fat (IMF) is a critical determinant of sheep quality, directly influencing its flavor, tenderness, and juiciness. Although deep learning offers a promising avenue for non-destructive prediction, research has predominantly centered on pork, leaving sheep quality assessment underexplored and highlighting a critical scarcity of public, large-scale multimodal datasets. To overcome the insufficient representational power of single-modality approaches (e.g., B-mode ultrasound images), this paper makes two primary contributions. First, we construct and release a comprehensive multimodal sheep dataset, containing 1,728 samples of ultrasound images, corresponding attributes, and ground-truth IMF values. Second, we propose DB-KAN, a novel dual-branch regression network designed to leverage this rich data. DB-KAN features a Convolutional Neural Network (CNN) branch to extract spatial features from ultrasound images and a Transformer branch to process structured attributes like backfat thickness, eye muscle depth, and eye muscle area measured at the 12th/13th rib site. This dual-branch architecture effectively captures heterogeneous information. Crucially, the decoder innovatively incorporates a KAN-Based regression head (KBRH), which efficiently fuses these multimodal features for a precise final prediction. Experiments on our dataset, partitioned 8:1:1 for training, validation, and testing, demonstrate that DB-KAN achieves state-of-the-art performance. Ablation studies further validate the indispensable roles of both the dual-branch design and the KAN-based fusion strategy.

## Introduction

The establishment of a scientific and efficient system for assessing sheep quality remains a formidable challenge in meat science. Among the key metrics, Intramuscular Fat (IMF) is universally recognized as the core determinant of both economic value and consumer-perceived eating quality^1,2^. Distributed as fine “marbling” within muscle, IMF content enhances tenderness and juiciness while its lipid oxidation during cooking generates the volatile compounds essential for sheep’s unique flavor and color^3,4^. Consequently, developing rapid, accurate, and non-destructive methods for IMF detection is of paramount importance for advancing genetic breeding, enabling precision farming, and establishing reliable grading systems^5,6^.

The pursuit of such methods is driven by the severe limitations of traditional techniques. While Soxhlet extraction is considered the “gold standard” for accuracy, it is destructive, time-consuming, and chemically complex, making it impractical for modern industry. Non-destructive alternatives such as Near-Infrared (NIR) spectroscopy have been explored; however, NIR typically requires samples collected post-slaughter, and estimating intramuscular fat (IMF) in live animals remains challenging. Moreover, its predictive accuracy is often compromised by the physical state of the sample and the fragility of calibration models, limiting widespread adoption^5,7,8^. In the wider phenotyping context of monitoring livestock fat deposition, significant strides have been made using optical sensors; for example, Zin et al. and Imamura et al. have successfully employed 3D cameras to estimate Body Condition Scores (BCS) in cattle through regression analysis^9,10^. While these approaches proficiently evaluate external anatomical traits, they are unable to penetrate tissue to reveal internal composition. Consequently, the accurate estimation of intramuscular fat (IMF) in living animals remains a persistent challenge.

**Fig. 1:**
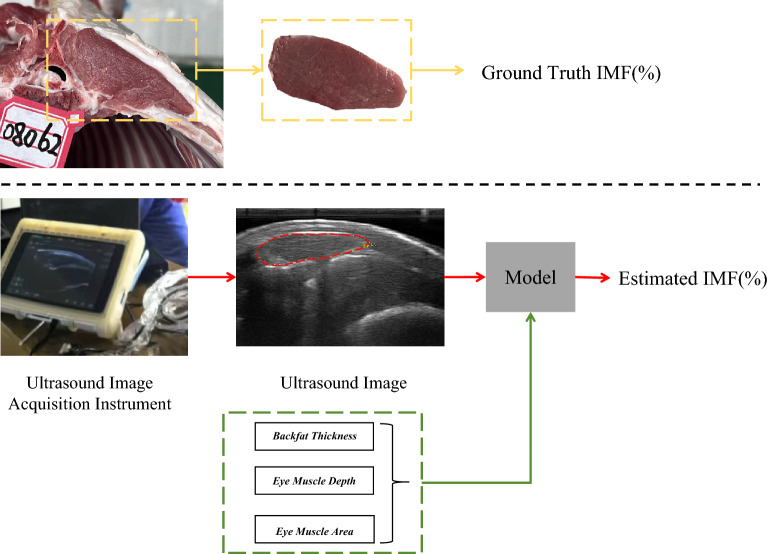
Comparison of our multi-modality method (the steps marked in red and green lines) with two main categories of existing approaches: conventional methods for IMF measurement (top row), and prediction methods based on single-modality (unimodal) B-mode ultrasound images (the steps marked in red line).

To bridge this gap, Real-time Ultrasound (RTU) has emerged as a superior non-destructive tool, offering a powerful combination of being non-invasive, low-cost, portable, and capable of real-time imaging. Research has confirmed that livestock IMF can be effectively predicted by analyzing textural features in ultrasound images alongside biological parameters^11^. As indicated by the red line in the second row of Fig. 1, they feed the B-mode ultrasound image into a model to regress the IMF result. However, the statistical models traditionally used for this task, such as Partial Least Squares Regression (PLSR) and Support Vector Machines (SVM)^12,13^, face a significant bottleneck: they struggle to extract deep, abstract features from high-dimensional ultrasound images and fail to capture their complex non-linear relationships. To overcome this limitation, combining image-derived texture features with established physiological metrics (such as backfat thickness and related attributes) helps to capture a more complete picture of IMF variation.

The advent of deep learning has triggered a paradigm shift in IMF prediction. In the more mature domain of porcine research, advanced Convolutional Neural Networks (CNNs) have achieved high precision^14,15^, and pioneering studies have demonstrated that a multimodal approach–fusing ultrasound images with physiological data–is a highly effective pathway to improving accuracy^16^ . While progress has been made in sheep-specific models like EIMFS^17^, which optimized feature extraction from single images , this highlights a critical research gap. Despite the proven success of multimodal strategies in porcine studies, advanced prediction models for sheep remain confined to single-modality ultrasound data^17,18^. This is a significant oversight, as it ignores information-rich attributes–such as weight, subcutaneous fat thickness, and eye muscle area, that are physiologically integral to IMF formation^19–21^. This reliance on a single modality results in incomplete feature representation, which inevitably compromises the model’s generalization ability and robustness in real-world scenarios. Furthermore, single-modality approaches, relying solely on image features, often neglect crucial, well-established physiological parameters like backfat thickness and eye muscle depth, which are known indicators of carcass composition. Therefore, introducing and validating a multimodal learning strategy for sheep IMF prediction is not merely an incremental improvement but a necessary evolution for the field.

To address this gap, this paper breaks new ground from both a data and a modeling perspective. Our contributions are threefold:

1. We constructed and present a dataset of 1,728 samples, each containing an ultrasound image, corresponding attributes, and a ground-truth IMF value. This resource fills a critical void and provides a benchmark for future research.
2. We designed a joint architecture integrating a CNN branch, to capture fine-grained spatial features from images, with a Transformer branch, to learn correlations within physiological data. This addresses the core limitation of information underutilization in single-modality methods.
3. We innovatively introduce a KAN-Based regression head (KBRH) based on the Kolmogorov-Arnold Network (KAN)^22^. Experiments confirm that this KAN-based module models the complex interactions between cross-modal features more effectively than traditional fusion mechanisms, verifying its superior performance.

## Materials and methods

### Animals and phenotypic measurements

A total of 300 domestic sheep (Ovis aries), representing six distinct populations, were included in this study. The sourcing was as follows: 24 Barchuk and 28 Dolang sheep were obtained post-mortem from a licensed commercial slaughterhouse in Marbashi County. Another 24 Chinese Merino sheep were housed at the research facility of the Institute of Animal Biotechnology in Urumqi. Additionally, 16 Dorper $\times$ Barchuk crossbred sheep were maintained at the Institute of Animal Science facility in Bohu County, and the remaining 98 Kazakh sheep and 102 Texel $\times$ Kazakh crossbred sheep were housed at the Institute of Animal Biotechnology facility in Zhaosu County. Critically, all animals were raised for routine meat production independently of this research, aligning with the ethical principle of Replacement by utilizing tissue that would otherwise be a byproduct.

For the experimental procedures, B-mode ultrasound images were non-invasively acquired from the animals prior to slaughter. Subsequently, tissue samples from the Longissimus Dorsi muscle were collected post-mortem. These samples were used to determine intramuscular fat content. The collection of slaughterhouse-derived samples was conducted in a manner that did not alter standard operational procedures, thus refining the process to avoid any additional impact on the animals. The determination of intramuscular fat content was conducted via the manual Soxhlet extraction method followed by the national food safety standards for the determination of fat in foods(GB5009.6-2016), and was completed by Agricultural Product Quality Inspection and Testing Center (Urumqi) of the Ministry of Agriculture and Rural Affairs. The entire study protocol, including all animal-related procedures, was rigorously reviewed and formally approved by the Animal Ethics Committee of the Xinjiang Uygur Autonomous Region Academy of Animal Science (Approval Number: 202390016).

### Dataset preparation

**Fig. 2:**
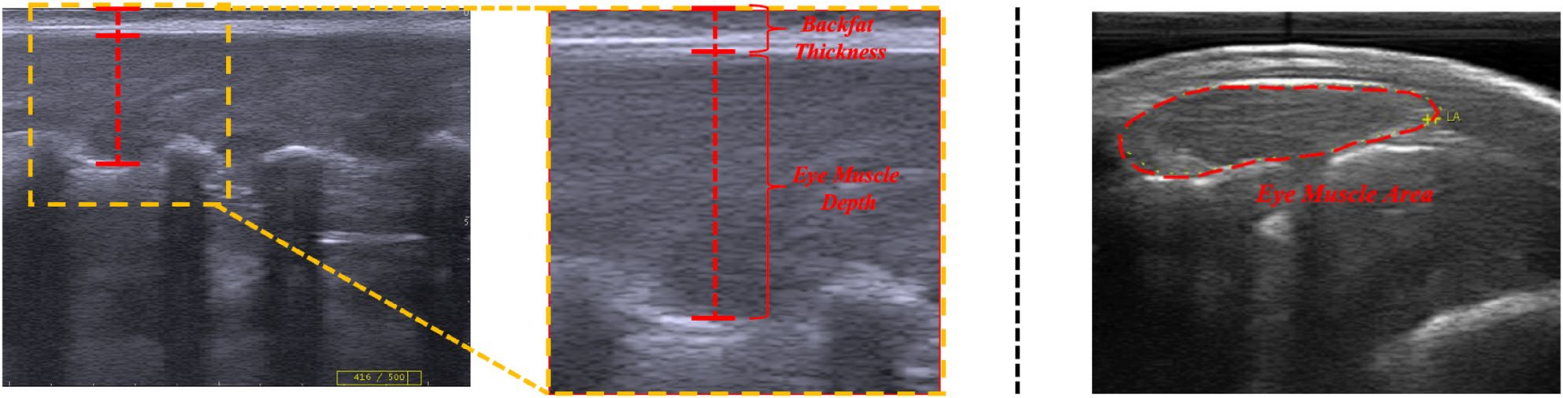
Schematic of numerical feature extraction from ultrasound images. The left figure shows the annotation method for backfat thickness and eye muscle depth, and the right figure shows the annotation method for eye muscle area.

**Table 1:** Summary of collected sheep samples by population. ($\times$ denotes a crossbred).

Population	Quantity	IMF Content Range (%)	No. of B-mode Images
Kazakh	98	0.3–6.1	656
Texel $\times$ Kazakh	102	0.5–4.0	663
Barchuk	24	0.5–3.0	145
Dolang	28	1.0–7.8	188
Chinese Merino	24	0.2–2.9	102
Dorper $\times$ Barchuk	16	1.5–4.8	128

To ensure a comprehensive validation of the proposed method’s efficacy, an extensive dataset was established, comprising samples from a total of 300 sheep across six distinct populations. These populations encompassed purebred Kazakh, Dolang, Barchuk, and Chinese Merino sheep, along with Texel $\times$ Kazakh and Dorper $\times$ Barchuk crossbreds. This diverse and large-scale dataset provides robust support for a thorough investigation into the correlation between in vivo B-mode ultrasound imagery and IMF content. A detailed breakdown of the populations and their respective sample sizes is summarized in Table 1.

During image acquisition, the sheep were properly restrained, either manually by multiple operators or using a restraint cradle, to ensure the animal remained calm and stable throughout the procedure. An area of approximately 10 cm on the left side of the sheep’s back, between the 12th and 13th ribs, was shaved. The shaved area was slightly larger than the footprint of the ultrasound probe to eliminate interference from wool on image quality. To ensure effective transmission of the ultrasound signal, a liberal amount of coupling gel was applied evenly to both the probe and the measurement area on the sheep’s back to prevent air interference.

Subsequently, the operator placed the probe parallel to the spine and moved it slowly over the measurement area to capture at least five images clearly showing the eye muscle depth and backfat thickness. In these longitudinal images, Backfat Thickness (BFT) was defined as the thickness of the subcutaneous fat layer, while Eye Muscle Depth (EMD) referred to the maximum vertical depth of the longissimus dorsi muscle. As shown in the left half of Fig. 2, we obtained these two values in each longitudinal image through manual annotation and then calculated the average of 5-6 images to serve as the final characteristic value. Immediately after, the probe was repositioned perpendicular to the spine and moved over the same area to capture at least five images showing a clear and complete cross-section of the eye muscle. As shown in the right half of Fig. 2, Eye Muscle Area (EMA) was then measured by tracing the entire outline of the eye muscle in this cross-sectional view. Similarly, we took the average of the these results to serve as the final characteristic value. The physiological traits, including backfat thickness, eye muscle depth, and eye muscle area, were measured manually using the electronic calipers and area measurement tools integrated into the ultrasound system’s software. These measurements were performed by trained technicians directly on the frozen B-mode images at the time of scanning to ensuring precise anatomical landmark identification.

**Fig. 3:**
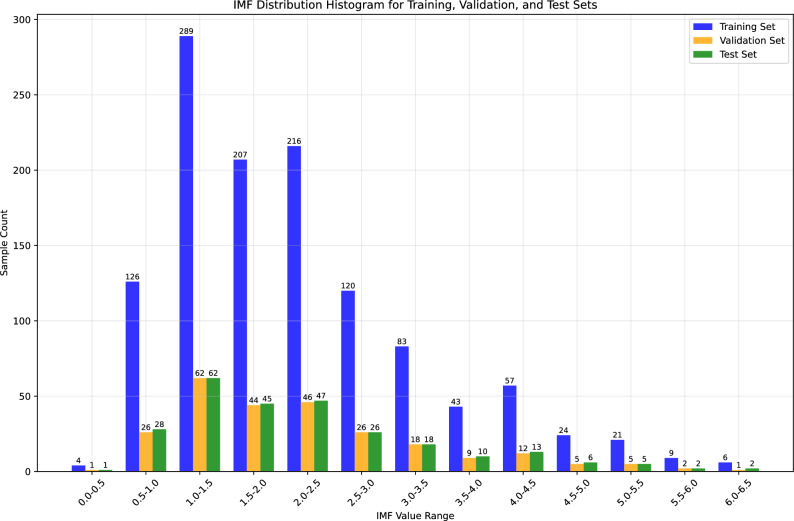
The IMF distribution histogram.

Following the completion of image acquisition, the sheep were slaughtered as scheduled. Immediately post-slaughter, each carcass was tagged with an identifier matching the sheep’s ear tag to ensure a one-to-one correspondence between the images and tissue samples. A sample of the longissimus dorsi muscle, weighing approximately 20 grams, was excised from the left side of the carcass at the position of the 13th last rib. The samples were stored under refrigeration before being transported to the laboratory, where the Intramuscular Fat (IMF) content was determined using the Soxhlet extraction method. The resulting IMF values served as the ground-truth labels for training and evaluating the image-based prediction model.

As shown in Table 1, a total of 1,882 raw samples were initially collected from six sheep populations. To ensure model robustness, strict quality control was applied: 154 samples were excluded due to artifacts caused by acquisition devices, image blurring, or missing/abnormal physiological data. Consequently, 1,728 valid, high-quality samples were retained for subsequent experiments. This curated dataset was then divided into 1,205 training samples, 257 validation samples, and 265 testing samples, respectively. The IMF distribution histogram is presented in Fig. 3. After excluding ultrasound images with artifacts attributable to the acquisition device, the curated dataset was subsequently divided. This process yielded a final distribution of 1,205 training samples, 257 validation samples, and 265 testing samples, respectively. The IMF distribution histogram is shown in Fig. 3. A complete training sample is composed of a set of features: the ultrasound image, the measured values for backfat thickness, eye muscle depth, and eye muscle area, and the target label, defined as the IMF value from the corresponding side.

### Model architecture

#### Overview of the proposed Dual-Branch KAN (DB-KAN) network

**Fig. 4:**
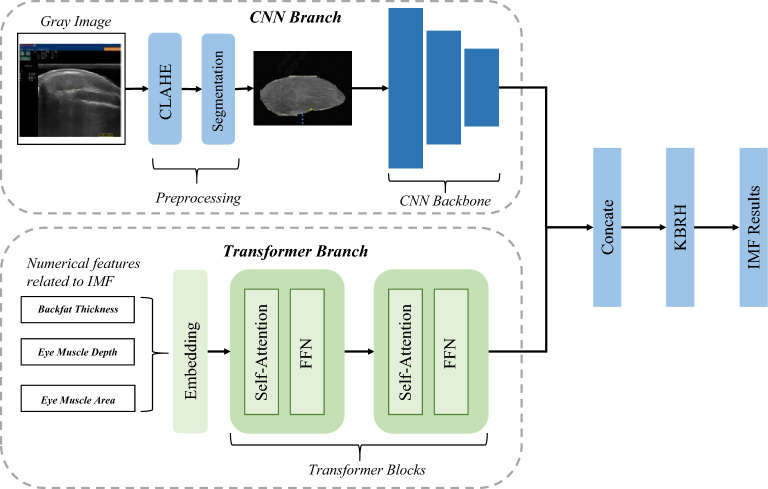
Overall framework of DB-KAN network.

The overall architecture of the proposed model is illustrated in Fig. 4. The Convolutional Neural Network (CNN) branch learns spatial features from B-mode ultrasound grayscale images, while the Transformer branch processes attribute data such as fat thickness and loin muscle dimensions. This dual-branch encoder architecture fully leverages the spatial features of images and complementary attribute data of sheep, addressing the limited representation capacity of single-modal approaches through heterogeneous feature representation. Furthermore, in the decoder section, a Kolmogorov-Arnold Network (KAN) layer is employed to achieve efficient fusion of multimodal features, ultimately generating the intramuscular fat (IMF) prediction results. We introduce each module in Fig. 4 in detail in the following sections.

#### CNN barnch

The CNN branch serves to capture detailed spatial information within the ultrasound images of sheep. Initially, the original B-mode ultrasound images are converted from RGB to single-channel grayscale (pixel values 0-255). This conversion not only significantly reduces computational complexity but also effectively suppresses potential device-related noise in color imaging, thereby accentuating key textural features. Subsequently, the Contrast Limited Adaptive Histogram Equalization (CLAHE)^23^ algorithm is applied to enhance local image contrast. Compared to traditional global histogram equalization, CLAHE^23^ operates by dividing the image into small grids (8$\times$8 in this study) and limiting the contrast enhancement in each region (clip limit = 2.0). This method effectively improves the subtle textural contrast between muscle fibers and adipose areas while mitigating the issues of noise amplification and artifact creation often caused by excessive local enhancement, thus improving the robustness of subsequent feature extraction.

Then, the pre-processed image is fed into a pre-trained Yolo-v8^24^ segmentation network, which captures multi-scale contextual information for precise segmentation of the eye muscle region (the ROI), generating a binary mask. This mask is then applied to the image via element-wise multiplication to eliminate non-target background pixels (such as the subcutaneous fat layer and fascia). This process ensures that feature extraction focuses exclusively on the anatomical region most relevant to IMF content.

Finally, we employ a classic ResNet^25^ as the feature extraction backbone. By introducing residual shortcuts, ResNet^25^ effectively mitigates the gradient vanishing problem in deep networks, enabling the model to learn higher-order semantic features by stacking more layers. To balance computational efficiency and hardware constraints, we selected the lightweight ResNet-18^25^ architecture. We removed its original global average pooling and fully-connected layers, utilizing its core convolutional modules to construct a feature pyramid.

#### Transformer branch

Existing methods^15,17^ predominantly rely on a single modality of ultrasound grayscale information, overlooking the auxiliary value provided by key physiological attributes such as population, body weight, and muscle dimensions. This results in an insufficient feature representation and limits the model’s predictive accuracy. To address this limitation, our study enriches the model’s input by incorporating additional numerical features that are strongly correlated with Intramuscular Fat (IMF), namely backfat thickness, eye muscle depth, and eye muscle area. While previous work^16^ have considered using multi-modal information, it often relied on a simplistic fusion strategy. This typically involved expanding the numerical features to match the spatial dimensions of the image, concatenating them with the image tensor, and then feeding the combined input into a Convolutional Neural Network (CNN). However, due to the inherent local receptive fields of convolution operations, such an approach is ineffective at capturing the global, non-spatial interactions between different numerical features (e.g., backfat thickness and eye muscle area). Consequently, crucial physiological correlations within this structured data are lost during the process.

To effectively extract features from these multi-source and heterogeneous data, we deliberately adopted a Transformer-based architecture^26^ for the physiological attribute branch, rather than the conventional Multi-Layer Perceptron (MLP). Although initially introduced for Natural Language Processing (NLP), the core principles of the Transformer–specifically the self-attention mechanism–are equally effective for capturing complex relationships within structured numerical data. While traditional MLPs process tabular data as a flat vector and interact with features implicitly through fixed weights, often overlooking intricate dependencies, the Transformer employs a Multi-Head Self-Attention mechanism to explicitly model feature interactions. This allows the network to dynamically prioritize critical physiological indicators (e.g., the synergistic effect of body weight and backfat thickness on fat deposition) based on their correlation with the target IMF. Furthermore, the multi-head design decouples the differentiated contributions of various feature subspaces, significantly enhancing robustness. Crucially, this approach projects discrete attribute values into a high-dimensional embedding space, ensuring that the tabular representations are semantically aligned with the high-level abstract features extracted by the CNN branch, thereby bridging the semantic gap and facilitating more effective multimodal fusion.

Specifically, the collected numerical features first undergo dimensionality expansion through an embedding layer:

1 $$\begin{aligned} \textbf{H}_{\text {emb}} = \textbf{X} \cdot \textbf{W}_{\text {emb}}^T + \textbf{b}_{\text {emb}} \end{aligned}$$

where $\textbf{X}\in \mathbb {R}^{B \times N_{\text {feat}}}$ is the input numerical features, *B* is the batch size and $N_{\text {feat}}=3$ is the number of original numerical features. $\textbf{W}_{\text {emb}} \in \mathbb {R}^{D_{\text {emb}} \times N_{\text {feat}}}$ is the learnable weight matrix of the embedding layer, with $D_{\text {emb}}=64$ being the embedding dimension. $\textbf{b}_{\text {emb}} \in \mathbb {R}^{D_{\text {emb}}}$ is the learnable bias vector of the embedding layer. $\textbf{H}_{\text {emb}} \in \mathbb {R}^{B \times D_{\text {emb}}}$ is the resulting output embedding. In essence, this operation maps each sample’s $N_{\text {feat}}$-dimensional feature vector into a richer $D_{\text {emb}}$-dimensional vector, preparing it for processing by the subsequent Transformer^26^ blocks.

$\textbf{H}_{\text {emb}}$ was then fed into two consecutive Transformer^26^ blocks. Within each block, the multi-head self-attention mechanism adaptively learns the importance weight of each physiological attribute for Intramuscular Fat (IMF) prediction by computing a similarity matrix between features (i.e., the Query-Key matrix). Meanwhile, residual connections and layer normalization ensure stable gradient propagation throughout the network. This design allows the model to overcome the linear combination assumption inherent in traditional statistical methods, enabling it to fully explore the deep, interactive patterns within the multi-dimensional data.

#### KAN-Based regression head for IMF prediction

We introduce a specialized KAN-Based regression head (KBRH) to effectively integrate multi-modal features for the prediction of IMF. The selection of the Kolmogorov-Arnold Network (KAN) over traditional Multi-Layer Perceptrons (MLPs) or MLP-Mixers is theoretically grounded in the Kolmogorov-Arnold representation theorem, which states that any multivariate continuous function can be represented as a superposition of continuous univariate functions. While MLPs approximate this using fixed activation functions (e.g., ReLU) on neurons and linear weights on edges, KANs innovate by placing learnable non-linear activation functions–parameterized by B-splines–directly on the edges. This architectural distinction provides a critical advantage in multimodal fusion tasks, where the relationship between heterogeneous inputs (visual patterns from CNNs and physiological metrics from Transformers) and the IMF value is highly non-linear and complex. The B-spline basis in KAN allows the network to fine-tune local variances in the feature space adaptively without disrupting the global approximation, offering a significantly more flexible curve-fitting capability than the global linear transformations typical of MLPs. Furthermore, unlike MLP-Mixers, which rely on dense matrix multiplications that can obscure feature interactions, KANs decompose the high-dimensional regression problem into a composition of 1D functions. This structure enables the model to achieve superior expressiveness with greater parameter efficiency, effectively mitigating the risk of overfitting on limited biological datasets while maintaining robust performance in fusing dual-branch features.

The input to this module is a concatenated feature vector, comprising high-level spatial details extracted from ultrasound images by ResNet-18^25^, and quantitative metrics derived from the sheep’s physical attributes by Transformer^26^ blocks.

**Fig. 5:**
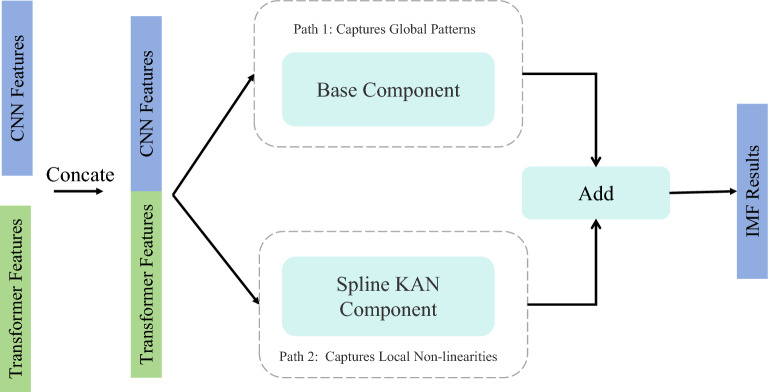
Illustration of the KAN-Based regression head for IMF prediction.

The architecture of the KAN module is inspired by Kolmogorov-Arnold Networks (KANs)^22^, but it is uniquely designed with a dual-pathway structure to process the fused features. Let the input to the module be a data batch, represented by the matrix $\textbf{X} \in \mathbb {R}^{B \times D_{in}}$, where *B* is the batch size and $D_{in}$ is the total dimension of the input features. For any single sample $\textbf{x} \in \mathbb {R}^{D_{in}}$ within the batch, it is formed by concatenating two feature vectors:

2 $$\begin{aligned} \textbf{x} = [\textbf{x}_{img} \Vert \textbf{x}_{attr}] \end{aligned}$$

where $\textbf{x}_{img} \in \mathbb {R}^{D_{img}}$ is the feature vector extracted from the ultrasound image and flattened into one dimension. $\textbf{x}_{attr} \in \mathbb {R}^{D_{attr}}$ is the one-dimensional feature vector of the sheep’s physical attributes. $\Vert$ denotes the concatenation operation. The total input dimension is $D_{in} = D_{img} + D_{attr}$.

The function of the whole module can be described as a mapping $f: \mathbb {R}^{D_{in}} \rightarrow \mathbb {R}^{D_{out}}$. The module’s output is a prediction vector $\textbf{y} \in \mathbb {R}^{D_{out}}$. In this specific application, $D_{out}=1$, representing the predicted Intramuscular Fat (IMF) value. As shown in Fig. 5 , the module consists of two parallel components: a base component module (BCM, $f_{base}$) and a spline-based KAN component (SKC, $f_{spline}$).

3 $$\begin{aligned} \textbf{y} = f(\textbf{x}) = f_{base}(\textbf{x}) + f_{spline}(\textbf{x}) \end{aligned}$$

The BCM functions analogously to a standard fully-connected layer and is designed to capture global, linear, and simple non-linear relationships within the features. Its mathematical expression is:

4 $$\begin{aligned} f_{base}(\textbf{x}) = \textbf{W}_{base} \cdot \sigma _b(\textbf{x}) \end{aligned}$$

where $\textbf{x} = (x_1, x_2, \dots , x_{D_{in}})$ is the input feature vector. $\sigma _b$ is a fixed, element-wise base activation function, such as SiLU (Sigmoid Linear Unit)^27^, where $\sigma _b(z) = z \cdot \text {sigmoid}(z)$. $\textbf{W}_{base} \in \mathbb {R}^{D_{out} \times D_{in}}$ is the learnable weight matrix of the base component. The *j*-th component of its output vector, $(f_{base}(\textbf{x}))_j$, can be written as:

5 $$\begin{aligned} (f_{base}(\textbf{x}))_j = \sum _{i=1}^{D_{in}} (\textbf{W}_{base})_{ji} \cdot \sigma _b(x_i) \end{aligned}$$

The SKC is the core of our module, which replaces fixed activation functions with learnable B-spline functions to capture complex, local non-linearities in the data. We first model the relationship between the input $\textbf{x}$ and the output $\textbf{y}$ as a sum of multiple learnable univariate activation functions, denoted as $\phi _{ji}$:

6 $$\begin{aligned} (f_{spline}(\textbf{x}))_j = \sum _{i=1}^{D_{in}} \phi _{ji}(x_i) \end{aligned}$$

where $\phi _{ji}(x_i)$ represents the contribution of the input feature $x_i$ to the output component $y_j$, and it is itself a learnable function. Each learnable activation function $\phi _{ji}$ is parameterized by a linear combination of B-spline basis functions:

7 $$\begin{aligned} \phi _{ji}(x_i) = \sum _{k=1}^{K_{spline}} c_{jik} \cdot B_{i,k}(x_i) \end{aligned}$$

where $B_{i,k}(x_i)$ is the *k*-th B-spline basis function defined on an adaptive grid associated with input feature $x_i$. The value of these basis functions is determined by the input $x_i$ and the grid knot locations, while their functional form is fixed. $c_{jik}$ are the learnable spline coefficients. These are the parameters that the model optimizes during training, forming a weight tensor $\textbf{C} \in \mathbb {R}^{D_{out} \times D_{in} \times K_{spline}}$. $K_{spline}$ is the number of B-spline basis functions, which is equal to $grid\_size + spline\_order$. By substituting the definition of $\phi _{ji}$, we obtain the complete expression for the output of the spline component:

8 $$\begin{aligned} (f_{spline}(\textbf{x}))_j = \sum _{i=1}^{D_{in}} \left( \sum _{k=1}^{K_{spline}} c_{jik} \cdot B_{i,k}(x_i) \right) \end{aligned}$$

Finally, the outputs from the two components are added element-wise to produce the final output vector $\textbf{y}$. The *j*-th component of the output, $y_j$, is given by:

9 $$\begin{aligned} y_j = \underbrace{\sum _{i=1}^{D_{in}} (\textbf{W}_{base})_{ji} \cdot \sigma _b(x_i)}_{\text {Global Patterns}} + \underbrace{\sum _{i=1}^{D_{in}} \sum _{k=1}^{K_{spline}} c_{jik} \cdot B_{i,k}(x_i)}_{\text {Local Non-linear Details}} \end{aligned}$$

This hybrid design allows the model to simultaneously learn both simple, global patterns and intricate, localized non-linearities from the heterogeneous data sources. Furthermore, the module incorporates an adaptive grid update mechanism, which dynamically adjusts the spline knots based on the input data distribution, enhancing its representational power. Regularization techniques are also applied to the spline coefficients to encourage sparsity, thereby improving model interpretability and preventing overfitting. Ultimately, this module transforms the fused multi-modal feature vector into a precise, final prediction of the sheep’s IMF.

## Results

### Experiment setting

To enhance the model’s robustness to geometric transformations and illumination variations, we employed a series of online data augmentation strategies, subjecting each B-mode ultrasound image to random rotations (within a $\pm {15}^{\circ }$ range), random horizontal flips ($p=0.5$), and random brightness adjustments ($\beta$ sampled from [0.8, 1.2]) to diversify the training data and mitigate overfitting. To ensure compatibility with the feature extraction backbone, raw ultrasound images were resized to 224$\times$224 pixels, which is the standard input dimension required by the pre-trained ResNet18 architecture. This resizing was performed using bilinear interpolation, after which the images were normalized using a pre-calculated mean ($\mu =0.4427$) and standard deviation ($\sigma =0.1844$) derived from the training set.

All experiments were conducted on a single NVIDIA RTX 2080Ti GPU using the PyTorch framework. An efficient data loading pipeline was implemented with a batch size of 64. For optimization, we used the Mean Squared Error (MSE) as the loss function and the Adam optimizer with an initial learning rate of $3\times 10^{-4}$ ($\beta _1=0.9$, $\beta _2=0.999$). The model was trained for 500 epochs, with performance evaluated on a validation set after each epoch. Finally, the model weights yielding the best validation performance were selected for final evaluation on the independent test set to report the final metrics.

### Performance evaluation metrics

Follow the previous work, we adopt the coefficient of determination ($\mathbf {R^2}$)^28^, mean squared error (MSE), root mean squared error (RMSE)^29^, Spearman’s rank correlation coefficient ($\rho$)^30^, and Pearson correlation coefficient (*r*)^31^ for the IMF content regression network.

10 $$\begin{aligned} R^2= & 1 - \frac{\sum _{i=1}^{n} (y_i - \hat{y}_i)^2}{\sum _{i=1}^{n} (y_i - \bar{y})^2} \end{aligned}$$

11 $$\begin{aligned} \text {MSE}= & \frac{1}{n} \sum _{i=1}^{n} (y_i - \hat{y}_i)^2\end{aligned}$$

12 $$\begin{aligned} \text {RMSE}= & \sqrt{\text {MSE}} \end{aligned}$$

13 $$\begin{aligned} \rho= & 1 - \frac{6 \sum d_i^2}{n(n^2 - 1)} \end{aligned}$$

14 $$\begin{aligned} r= & \frac{\sum _{i=1}^{n} (x_i - \bar{x})(y_i - \bar{y})}{\sqrt{\sum _{i=1}^{n} (x_i - \bar{x})^2} \sqrt{\sum _{i=1}^{n} (y_i - \bar{y})^2}} \end{aligned}$$

The evaluation metrics used in this study are defined by the equations presented above, where $y_i$ represents the observed value, $\hat{y}_i$ represents the predicted value, $\bar{y}$ represents the mean of the observed values, $x_i$ and $y_i$ represent the observed values of two sets of data, respectively, $\bar{x}$ represents the mean value of the data, $d_i$ represents the sorted difference between the two sets of data, and *n* represents the number of data points.

### Comparison with other methods

In this section, we conduct a comprehensive evaluation of our proposed method against a variety of unimodal and multimodal baselines. The unimodal baselines are divided into two categories: 1) Mainstream computer vision backbones, including AlexNet^32^, VGG16^33^, ResNet18^25^, ResNet50^25^, MobileNet^34^, and ViT^35^, which serve to evaluate general feature extraction capabilities. 2) Recent models named EIMFS^17^ and PIMPF^15^, which are developed specifically for IMF prediction, included to ensure a direct and thorough comparison. For the multimodal evaluation, we benchmark our approach against the recent state-of-the-art method, BLMNet^16^. As the official code for these methods is not publicly available, we have meticulously re-implemented their models by strictly following the descriptions in their respective papers. To ensure a fair comparison, the CNN backbone for all methods we re-implemented was standardized to ResNe18. The quantitative results of our comparative analysis are presented in Table 2. The findings clearly indicate the superior performance of our proposed method over all baseline models.

**Table 2:** Comparison with single-modality (SM, image) and multi-modal (MM, image-attribute) approaches. The “Year” column indicates the publication time of the model or method.

Method	Model	Year	$R^2$ $\uparrow$	MSE $\downarrow$	RMSE $\downarrow$	$\rho$ $\uparrow$	*r* $\uparrow$
SM	AlexNet	2012	0.1421	0.8296	0.9108	0.1019	0.0059
	VGG16	2014	0.3347	0.6433	0.8021	0.6268	0.6573
	ResNet18	2016	0.5407	0.4441	0.6664	0.7446	0.7553
	ResNet50	2016	0.4621	0.5201	0.7212	0.6852	0.6801
	MobileNet	2017	0.4131	0.5675	0.7533	0.7881	0.7257
	ViT-B16	2021	0.5371	0.4476	0.6691	0.8168	0.7786
	PIMPF^#^	2024	0.5922	0.3945	0.6281	0.8131	0.8186
	EIMFS^#^	2025	0.5993	0.3875	0.6225	0.8001	0.7808
MM	BLMNet^#^	2024	0.7625	0.3216	0.5671	0.8224	0.8749
	**Ours**	**2025**	**0.8273**	**0.2339**	**0.4836**	**0.8728**	**0.9156**

^#^ indicates that these models were re-implemented by us. All results represent the average of 5-fold cross-validation (Mean ± Std).

Among the single-modality (SM) methods, we observe a consistent trend where more advanced network architectures yield better results. For instance, modern backbones like ResNet50 ($\text {R}^{2}=0.4621$, RMSE=0.7212) and MobileNet ($\text {R}^{2}=0.4131$, RMSE=0.7533) significantly outperform earlier models such as AlexNet ($\text {R}^{2}=0.1421$, RMSE=0.9108) and VGG16 ($\text {R}^{2}=0.3347$, RMSE=0.8021). The specialized baseline models, EIMFS and PIMPF, which were re-implemented for this study, demonstrate strong performance, with EIMFS achieving the highest results among all SM methods ($\text {R}^{2}=0.5993$, RMSE=0.6225), followed by PIMPF ($\text {R}^{2}=0.5922$, RMSE=0.6281). Notably, ViT-B16 shows competitive performance with $\text {R}^{2}=0.5371$ and RMSE=0.6691. However, the multi-modal (MM) approaches show a marked improvement over the unimodal ones, underscoring the benefits of integrating auxiliary attribute data. Our proposed model, in particular, establishes a new state-of-the-art by surpassing the recent BLMNet method ($\text {R}^{2}=0.7625$, RMSE=0.5671). It achieves the best performance across all five evaluation metrics, with an $\text {R}^{2}$ of 0.8273, MSE of 0.2339, RMSE of 0.4836, Spearman correlation ($\rho$) of 0.8728, and Pearson correlation (*r*) of 0.9156. These results robustly validate the effectiveness of our model architecture in fusing multi-modal information, leading to a more accurate and reliable prediction.

### Ablation study

#### Comparative ablation study of single-modality and multi-modal approaches

**Table 3:** Comparative ablation study of single-modality and multi-modal approaches. (w/o means “without”).

Method	$R^2$ $\uparrow$	MSE $\downarrow$	RMSE $\downarrow$	$\rho$ $\uparrow$	*r* $\uparrow$
Original DB-KAN	**0.8273**	**0.2339**	**0.4836**	**0.8728**	**0.9156**
w/o CNN Branch	0.6799	0.4333	0.6583	0.7379	0.8302
w/o Transformer Branch	0.8027	0.2671	0.5168	0.8379	0.8961

We assessed the performance of individual modalities by respectively removing the CNN branch (for image modality) and Transformer branch (for attribute modality) in DB-KAN. The ablation study in Table 3 demonstrates the critical importance of multi-modal integration compared to single-modality configurations. The complete DB-KAN framework achieves optimal performance across all metrics ($R^2$: 0.8273, MSE: 0.2339, RMSE: 0.4836, $\rho$: 0.8728, *r*: 0.9156). Significant performance degradation occurs when removing individual modalities: Ablation of the CNN branch (image modality) causes the most severe deterioration, with $R^2$ plunging 17.8% to 0.6799 and MSE increasing 85.3% to 0.4333. In contrast, removing the Transformer branch (attribute modality) yields relatively moderate declines ($R^2$: 0.8027, -3.0%; MSE: 0.2671, +14.2%). These results confirm that while visual features processed by CNN constitute the dominant information source, the synergistic integration of attribute features through Transformer processing provides complementary benefits. The full multi-modal configuration delivers a 23.8% $R^2$ improvement over the best single-modality alternative, underscoring that neither modality alone can match the robustness and accuracy achieved through cross-modal fusion.

**Fig. 6:**
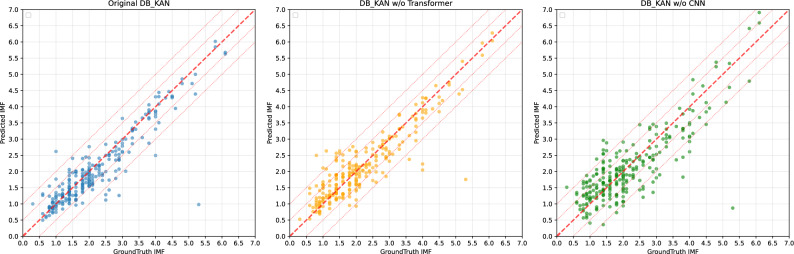
Comparison of prediction scatter plots for the original model and after modal ablation. The thick red dashed line (slope 1 through the origin) denotes the perfect prediction line, and the thin red dotted lines are parallel equal-error bands. Tighter clustering around the perfect line indicates higher accuracy, while points on the outer bands or beyond suggest larger errors or potential systematic bias.

Figure 6 illustrates the comparison of prediction scatter plots for the original DB-KAN model and after modal ablation. The scatter plot is constructed with the ground truth IMF values on the horizontal axis and the predicted IMF values on the vertical axis. The plot includes several reference lines: a central solid line (y=x) representing an ideal prediction, and dashed lines that delineate the $\pm {0.5}$ and $\pm {1.0}$ error boundaries. As observed from the scatter distribution, the predictions from the Original DB-KAN model align closely with the actual values. The data points are relatively concentrated, with the majority falling within the $\pm {1.0}$ error margin, indicating a high degree of prediction accuracy. In contrast, the DB-KAN w/o Transformer model, which has its attribute branch removed, exhibits more outliers and larger prediction deviations, particularly in the high-IMF value region ($> 5.0$). Similarly, after removing the CNN branch, the scatter distribution for the DB-KAN w/o CNN model becomes more dispersed, showing a decline in prediction accuracy in both the low-IMF ($< 1.0$) and high-IMF ($>5.0$) regions.

#### Comparative ablation study of the effectiveness Of KAN decoder

**Table 4:** Comparative ablation study of the effectiveness of KAN decoder. (w/o means “without”).

Method	$R^2$ $\uparrow$	MSE $\downarrow$	RMSE $\downarrow$	$\rho$ $\uparrow$	*r* $\uparrow$
Original DB-KAN	**0.8273**	**0.2339**	**0.4836**	**0.8728**	**0.9156**
w/o KAN	0.7477	0.3415	0.5844	0.7699	0.7668

**Fig. 7:**
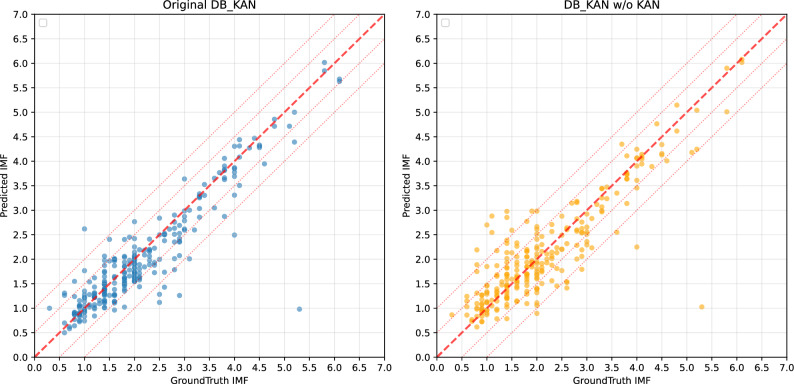
Comparison of prediction scatter plots for the original model and replaced the KAN-based regression prediction head in DB-KAN with standard fully-connected layer. The thick red dashed line (slope 1 through the origin) denotes the perfect prediction line, and the thin red dotted lines are parallel equal-error bands. Tighter clustering around the perfect line indicates higher accuracy, while points on the outer bands or beyond suggest larger errors or potential systematic bias.

We replaced the KAN-based regression prediction head in DB-KAN with standard fully-connected layers to evaluate the effectiveness of KAN for multimodal information fusion. The results in Table 4 conclusively validates the critical contribution of the KAN decoder to model performance, where removal of the KAN module (w/o KAN) triggers comprehensive performance degradation across all metrics. Prediction accuracy substantially deteriorates, with $R^2$ decreasing by 9.6% (0.8273 $\rightarrow$ 0.7477), MSE increasing by 46.0% (0.2339 $\rightarrow$ 0.3415, absolute $\Delta =0.1076$), and RMSE rising 20.9% to 0.5844, while correlation metrics simultaneously weaken as evidenced by an 11.8% reduction in Spearman’s $\rho$ (0.8728 $\rightarrow$ 0.7699) and a 16.2% decline in Pearson’s *r* (0.9156 $\rightarrow$ 0.7668). This significant performance gap underscores KAN’s indispensable role in feature decoding, as its interpretable spline-based mechanism enables superior nonlinear mapping capabilities that allow adaptive basis functions to capture complex data patterns unattainable by conventional decoding architectures. The consistent deterioration across both prediction accuracy ($R^2$ /MSE/RMSE) and feature correlation ($\rho$/*r*) metrics demonstrates that KAN’s mathematical formulation is fundamental to maintaining the model’s robustness and expressive power.

As observed from the generated scatter plots in Fig. 7, a clear distinction in predictive performance emerges. The left panel, representing the original DB-KAN model, shows data points highly concentrated around the diagonal line. This tight clustering indicates that for the majority of instances, the deviation between predicted and true values is minimal, signifying a high level of prediction accuracy. In contrast, the right panel, which illustrates the performance of the DB-KAN model without the KAN component, displays a considerably more dispersed distribution of points. The increased scatter and greater deviation from the diagonal line are indicative of a marked decline in predictive precision.

#### Comparative ablation study of the number Of transformer blocks

**Table 5:** Comparative ablation study of the number of transformer blocks.

Method	$R^2$ $\uparrow$	MSE $\downarrow$	RMSE $\downarrow$	$\rho$ $\uparrow$	*r* $\uparrow$
1 block	0.7815	0.2941	0.5423	0.8412	0.8894
**2 blocks (Ours)**	**0.8273**	**0.2339**	**0.4836**	**0.8728**	**0.9156**
3 blocks	0.8269	0.2345	0.4842	0.8725	0.9151

To verify the rationality of the network depth, we compared the performance of 1, 2, and 3 Transformer blocks. As shown in Table 5, a single block resulted in suboptimal performance due to limited non-linear modeling capacity. Increasing the depth to 3 blocks showed performance saturation, yielding results almost identical to the 2-block configuration without any significant improvement. Consequently, we selected the 2-block structure as the most parameter-efficient solution that ensures maximum expressiveness.

#### Comparison with SOTA methods with 5-fold cross-validation

**Table 6:** Performance comparison of single-modality (SM) and multi-modal (MM) approaches. Results are reported as Mean ± Std from 5-fold cross-validation.

Method	Model	Year	$R^2$ $\uparrow$	MSE $\downarrow$	RMSE $\downarrow$	$\rho$ $\uparrow$	*r* $\uparrow$
SM	AlexNet	2012	0.1150 ± 0.102	0.8562 ± 0.125	0.9251 ± 0.075	0.0850 ± 0.088	0.0025 ± 0.095
	VGG16	2014	0.3105 ± 0.085	0.6672 ± 0.091	0.8168 ± 0.062	0.6015 ± 0.070	0.6320 ± 0.068
	ResNet18	2016	0.5088 ± 0.065	0.4755 ± 0.068	0.6895 ± 0.052	0.7205 ± 0.055	0.7310 ± 0.050
	ResNet50	2016	0.4320 ± 0.071	0.5498 ± 0.075	0.7415 ± 0.058	0.6610 ± 0.061	0.6550 ± 0.058
	MobileNet	2017	0.3850 ± 0.078	0.5950 ± 0.082	0.7713 ± 0.060	0.7620 ± 0.065	0.7015 ± 0.068
	ViT-B16	2021	0.5125 ± 0.058	0.4715 ± 0.061	0.6866 ± 0.048	0.7950 ± 0.049	0.7520 ± 0.051
	PIMPF	2024	0.5680 ± 0.051	0.4182 ± 0.054	0.6465 ± 0.042	0.7910 ± 0.038	0.7955 ± 0.036
	EIMFS	2025	0.5750 ± 0.048	0.4110 ± 0.051	0.6410 ± 0.038	0.7785 ± 0.035	0.7590 ± 0.034
MM	BLMNet	2024	0.7385 ± 0.038	0.3450 ± 0.041	0.5873 ± 0.035	0.8015 ± 0.030	0.8520 ± 0.028
	**Ours**	**2025**	**0.8095 ± 0.024**	**0.2510 ± 0.029**	**0.5010 ± 0.025**	**0.8580 ± 0.021**	**0.9012 ± 0.019**

Table 6 presents the quantitative comparison using 5-fold cross-validation. As expected, the rigorous cross-validation scheme resulted in a slight decrease in overall metrics compared to typical single-split evaluations, reflecting the true difficulty of the dataset. Specifically, single-modality models struggled with the data heterogeneity, with ResNet18 achieving an $R^2$ of only $0.5088 \pm 0.065$. However, even under these more demanding conditions, our method demonstrated superior resilience. It achieved a mean $R^2$ of $0.8095 \pm 0.024$ and an RMSE of $0.5010 \pm 0.025$, significantly outperforming the second-best multi-modal baseline, BLMNet ($0.7385 \pm 0.038$). The relatively small standard deviation of our method further underscores its stability in handling diverse IMF distributions.

## Discussion

This paper proposes a dual-branch deep regression network named DB-KAN for sheep IMF prediction. To evaluate the effectiveness of the proposed multimodal approach while addressing the scarcity of multimodal research data in the field, this study first constructs a large-scale multimodal sheep dataset comprising 1,728 samples covering six major populations including Kazakh sheep and Dolang $\times$ Han hybrid sheep, with IMF content ranging from 0.2% to 7.8%. Each sample includes B-mode ultrasound images of the eye muscle region, values for backfat thickness, eye muscle depth, eye muscle area, and ground-truth IMF measurements obtained through Soxhlet extraction. This dataset establishes a reliable benchmark for sheep quality assessment research, overcoming the core bottleneck of “insufficient multimodal data” in existing studies.

The proposed DB-KAN network achieves precise prediction of sheep intramuscular fat (IMF) content by fusing multimodal data from B-mode ultrasound images and key physiological attributes (backfat thickness, eye muscle depth, eye muscle area). It innovatively incorporates a decoder based on the Kolmogorov-Arnold Network (KAN), addressing the representational limitations of traditional unimodal approaches in sheep IMF assessment.

In the CNN branch, B-mode ultrasound images undergo grayscale conversion and Contrast Limited Adaptive Histogram Equalization (CLAHE) preprocessing to enhance textural features. The eye muscle region (ROI) is localized using a U-Net segmentation network, and spatial features are extracted via ResNet18. In the Transformer branch, physiological attributes are encoded through an embedding layer, with self-attention mechanisms learning global correlations (e.g., synergistic effects between backfat thickness and body weight). The dual-branch architecture fully exploits complementary heterogeneous data, while the KAN decoder achieves nonlinear fusion of cross-modal features through adaptive spline functions, significantly enhancing prediction robustness. The DB-KAN model achieves breakthrough performance: $\text {R}^{2}=0.8273$, MSE=0.2339, RMSE=0.4836, comprehensively surpassing existing baseline models. Ablation experiments confirm the indispensability of both the dual-branch design and KAN decoder.

Currently, our team is developing automated segmentation and calculation methods for backfat thickness and eye muscle area based on ultrasound images. This aims to replace manual annotation with automated computation of backfat thickness and eye muscle area values, providing optimal input data for multimodal networks. This technology is expected to enable rapid, non-destructive, and precise detection of sheep backfat thickness, eye muscle area, and IMF content, thereby establishing a convenient one-click detection system.

Future work will focus on expanding the dataset’s scale and diversity to cover additional populations and broader IMF ranges, thereby enhancing model generalization capabilities, while also exploring transfer applications of this framework to predict other critical meat quality traits such as tenderness and water-holding capacity.

## Conclusions

This study presents DB-KAN, a novel dual-branch deep regression network for accurate intramuscular fat (IMF) prediction in sheep. To address the critical gap in multimodal research data for sheep quality assessment, we first constructed a large-scale multimodal dataset comprising 1,728 samples across six major populations (purebred Kazakh, Dolang, Barchuk, and Chinese Merino, as well as Texel $\times$ Kazakh and Dorper $\times$ Barchuk crossbreds), with IMF content spanning 0.2%–7.8%. Each sample integrates B-mode ultrasound images of the longissimus dorsi region, key physiological attributes (backfat thickness, eye muscle depth, and eye muscle area), and Soxhlet-extracted ground-truth IMF values. This dataset serves as an essential benchmark for future research, effectively overcoming the “insufficient multimodal data” bottleneck in the field.

The proposed DB-KAN framework achieves breakthrough performance by synergistically fusing heterogeneous data modalities. The CNN branch processes ultrasound images through grayscale conversion, CLAHE-based contrast enhancement, ROI segmentation, and ResNet18 spatial feature extraction. Simultaneously, the Transformer branch encodes physiological attributes via embedding layers and captures global feature correlations (e.g., backfat-weight synergies) through self-attention mechanisms. Crucially, the model introduces an innovative Kolmogorov-Arnold Network (KAN) decoder that leverages adaptive spline functions to achieve superior nonlinear fusion of cross-modal features, significantly enhancing prediction robustness. Experimental results show that DB-KAN achieves state-of-the-art performance with $\text {R}^{2}=0.8273$, MSE=0.2339, and RMSE=0.4836, comprehensively outperforming existing baselines. Ablation studies confirm the indispensability of both the dual-branch architecture and the KAN-based fusion strategy.

## Data Availability

All data generated and analysed during the current study are not publicly available, but the datasets mentioned above are available from the corresponding author on reasonable request.

## References

[1] Zhang, X., Liu, C., Kong, Y., Li, F. & Yue, X. Effects of intramuscular fat on meat quality and its regulation mechanism in tan sheep. *Front. Nutr.* **9**, 908355 (2022). 35967801 10.3389/fnut.2022.908355 PMC9366309

[2] Hopkins, D., Hegarty, R., Walker, P. & Pethick, D. Relationship between animal age, intramuscular fat, cooking loss, ph, shear force and eating quality of aged meat from sheep. *Aust. J. Exp. Agric.* **46**, 879–884 (2006).

[3] Love, J. D. & Pearson, A. Lipid oxidation in meat and meat products-a review. *J. Am. Oil Chem. Soc.* **48**, 547–549 (1971).

[4] Kosowska, M., A Majcher, M. & Fortuna, T. Volatile compounds in meat and meat products. *Food Sci. Technol.* **37**, 1–7 (2017).

[5] Wood, J. et al. Fat deposition, fatty acid composition and meat quality: A review. *Meat Sci.* **78**, 343–358 (2008). 22062452 10.1016/j.meatsci.2007.07.019

[6] Houghton, P. & Turlington, L. Application of ultrasound for feeding and finishing animals: A review. *J. Anim. Sci.* **70**, 930–941 (1992). 1564012 10.2527/1992.703930x

[7] Prieto, N., Pawluczyk, O., Dugan, M. E. R. & Aalhus, J. L. A review of the principles and applications of near-infrared spectroscopy to characterize meat, fat, and meat products. *Appl. Spectrosc.* **71**, 1403–1426 (2017). 28534672 10.1177/0003702817709299

[8] Pérez-Palacios, T., Ruiz, J., Martín, D., Muriel, E. & Antequera, T. Comparison of different methods for total lipid quantification in meat and meat products. *Food Chem.* **110**, 1025–1029 (2008). 26047297 10.1016/j.foodchem.2008.03.026

[9] Zin, T. T., Seint, P. T., Tin, P., Horii, Y. & Kobayashi, I. Body condition score estimation based on regression analysis using a 3d camera. *Sensors* **20**, 3705 (2020). 32630751 10.3390/s20133705 PMC7374283

[10] Imamura, S., Zin, T. T., Kobayashi, I. & Horii, Y. Automatic evaluation of cow’s body-condition-score using 3d camera. In *2017 IEEE 6th Global Conference on Consumer Electronics (GCCE)*, 1–2 (IEEE, 2017).

[11] Fabbri, G. et al. Application of ultrasound images texture analysis for the estimation of intramuscular fat content in the longissimus thoracis muscle of beef cattle after slaughter: A methodological study. *Animals* **11**, 1117 (2021). 33924697 10.3390/ani11041117 PMC8069777

[12] Fowler, S. M., Wheeler, D., Morris, S., Mortimer, S. I. & Hopkins, D. L. Partial least squares and machine learning for the prediction of intramuscular fat content of lamb loin. *Meat Sci.* **177**, 108505 (2021). 33773186 10.1016/j.meatsci.2021.108505

[13] Wu, J. et al. Non-destructive and efficient prediction of intramuscular fat in live pigs based on ultrasound images and machine learning. *Comput. Electron. Agric.* **234**, 110291 (2025).

[14] Liu, Z. et al. Pimfp: An accurate tool for the prediction of intramuscular fat percentage in live pigs using ultrasound images based on deep learning. *Comput. Electron. Agric.* **217**, 108552 (2024).

[15] Liu, Z. et al. Pimfp: An accurate tool for the prediction of intramuscular fat percentage in live pigs using ultrasound images based on deep learning. *Comput. Electron. Agric.* **217**, 108552 (2024).

[16] Liu, W., Liu, T., Zhang, J. & Wang, F. Two-stage multimodal method for predicting intramuscular fat in pigs. *Agriculture* **14**, 1843 (2024).

[17] Yang, Y. et al. Eimfs: Estimating intramuscular fat in sheep using a three-stage convolutional neural network based on ultrasound images. *Comput. Electron. Agric.* **233**, 110169 (2025).

[18] Shi, Y. et al. A review on meat quality evaluation methods based on non-destructive computer vision and artificial intelligence technologies. *Food Sci. Anim. Resour.* **41**, 563 (2021). 34291208 10.5851/kosfa.2021.e25 PMC8277176

[19] Budimir, K., Mozzon, M., Toderi, M., D’Ottavio, P. & Trombetta, M. F. Effect of breed on fatty acid composition of meat and subcutaneous adipose tissue of light lambs. *Animals* **10**, 535 (2020). 32210212 10.3390/ani10030535 PMC7143277

[20] Güngör, Ö. F., Özbeyaz, C., Ünal, N. & Akçapınar, H. The evaluation of the genotype and slaughter weight effect on meat quality and fatty acid profile from two native sheep. *Trop. Anim. Health Prod.* **55**, 116 (2023). 36928174 10.1007/s11250-023-03523-5

[21] de Lima Júnior, D. M. et al. Intrinsic factors affecting sheep meat quality: a review. *Rev. Colomb. Cienc. Pecu.* **29**, 03–15 (2016).

[22] Liu, Z. et al. Kan: Kolmogorov-arnold networks. *arXiv preprint* arXiv:2404.19756 (2024).

[23] Zuiderveld, K. *Contrast limited adaptive histogram equalization, 474–485* (Academic Press Professional Inc, USA, 1994).

[24] Jocher, G., Qiu, J. & Chaurasia, A. *Ultralytics YOLO* (2023).

[25] He, K., Zhang, X., Ren, S. & Sun, J. Deep residual learning for image recognition. In *Proceedings of the IEEE conference on computer vision and pattern recognition*, 770–778 (2016).

[26] Vaswani, A. et al. Attention is all you need. In *Proceedings of the Neural Inf. Process. Syst. ***30**, 5998–6008 (2017).

[27] Elfwing, S., Uchibe, E. & Doya, K. Sigmoid-weighted linear units for neural network function approximation in reinforcement learning. *arXiv preprint* arXiv:1702.03118 (2017). 10.1016/j.neunet.2017.12.012 29395652

[28] Gelman, A., Goodrich, B., Gabry, J. & Vehtari, A. R-squared for bayesian regression models. *Am. Stat. ***73**, 307–309 (2019).

[29] Hyndman, R. J. & Koehler, A. B. Another look at measures of forecast accuracy. *Int. J. Forecast.* **22**, 679–688 (2006).

[30] Spearman, C. The proof and measurement of association between two things. *Am. J. Psychol.* **39**, 1137–1150 (2010). 10.1093/ije/dyq191 21051364

[31] Edwards, A. Galton, karl pearson and modern statistical theory. In *Sir Francis Galton, FRS: The Legacy of His Ideas: Proceedings of the twenty-eighth annual symposium of the Galton Institute, London, 1991*, 91–107 (Springer, 1993).

[32] Krizhevsky, A., Sutskever, I. & Hinton, G. E. Imagenet classification with deep convolutional neural networks. In *Proceedings of the Neural Inf. Process. Syst.* **25**, 1097–1105 (2012).

[33] Simonyan, K. & Zisserman, A. Very deep convolutional networks for large-scale image recognition. *arXiv preprint* arXiv: 1409.1556 (2014).

[34] Howard, A. G. et al. Mobilenets: Efficient convolutional neural networks for mobile vision applications. *arXiv preprint* arXiv:1704.04861 (2017).

[35] Dosovitskiy, A. et al. An image is worth 16x16 words: Transformers for image recognition at scale. *arXiv preprint* arXiv:2010.11929 (2020).

